# Phytotoxicity and Phytotoxic Substances in *Calamus tenuis* Roxb.

**DOI:** 10.3390/toxins15100595

**Published:** 2023-10-02

**Authors:** Md. Mahfuzur Rob, Kawsar Hossen, Kaori Ozaki, Toshiaki Teruya, Hisashi Kato-Noguchi

**Affiliations:** 1 Department of Applied Biological Science, Faculty of Agriculture, Kagawa University, Miki 761-0795, Japan; mahfuzrob.hort@sau.ac.bd; 2 The United Graduate School of Agricultural Sciences, Ehime University, Matsuyama 790-8566, Japan; 3 Department of Horticulture, Faculty of Agriculture, Sylhet Agricultural University, Sylhet 3100, Bangladesh; 4 Department of Agriculture, Faculty of Science, Noakhali Science and Technology University, Noakhali 3814, Bangladesh; 5 Graduate School of Engineering and Science, University of the Ryukyus, 1 Senbaru, Nishihara, Okinawa 903-0213, Japan; kaorrira@gmail.com; 6 Faculty of Education, University of the Ryukyus, 1 Senbaru, Nishihara, Okinawa 903-0213, Japan; t-teruya@edu.u-ryukyu.ac.jp

**Keywords:** *Calamus tenuis*, phytotoxic substances, calamulactone, 3-oxo-α-ionone, bioherbicide

## Abstract

*Calamus tenuis* is a shrub species distributed across South Asia. It grows well in diversified habitats and tends to dominate plants in the surrounding environment. The phytotoxicity of *C. tenuis* and the action of its phytochemicals against other plant species could explain its dominant behavior. Compounds with phytotoxic activity are in high demand as prospective sources of ecofriendly bioherbicides. Therefore, we investigated the phytotoxicity of *C. tenuis*. Aqueous methanol extracts of this plant species significantly limited the growth of four test plant species, two monocots (barnyard grass and timothy), and two dicots (alfalfa and cress), in a dose- and species-dependent manner. Bio-directed chromatographic isolation of the *C. tenuis* extracts yielded two major active substances: a novel compound, calamulactone {(S)-methyl 8-(5-oxo-2,5-dihydrofuran-2-yl) octanoate}, and 3-oxo-α-ionone. Both of the identified compounds exerted strong growth inhibitory effects on cress and timothy seedlings. The concentrations of 3-oxo-α-ionone and calamulactone required to limit the growth of the cress seedlings by 50% (I_50_) were 281.6–199.5 and 141.1–105.5 µM, respectively, indicating that the effect of calamulactone was stronger with lower I_50_ values. Similarly, the seedlings of timothy also showed a considerably higher sensitivity to calamulactone (I_50_: 40.5–84.4 µM) than to 3-oxo-α-ionone (I_50_: 107.8–144.7 µM). The findings indicated that the leaves of *C. tenuis* have marked growth-inhibitory potential, and could affect surrounding plants to exert dominance over the surrounding plant community. Moreover, the two identified phytotoxic substances might play a key role in the phytotoxicity of *C. tenuis*, and could be a template for bioherbicide development. This paper was the first to report calamulactone and its phytotoxicity.

## 1. Introduction

Weed plants are highly adaptable and proliferate in a variety of habitats, disrupt agricultural crop development, and compromise ecosystem functioning. They can easily spread from their original habitats to other distant habitats [1]. Weeds are considered a serious menace to world agroecosystems, as they reduce the quality and productivity of agricultural crops [2]. They compete with agricultural crops for various growth resources, such as space, water, nutrients, light, and gases, as well as host pests and many diseases [3,4]. The reduction in crop yields caused by weeds varies greatly depending on crop type, weed control practices, composition of weeds, period of infestation, and different climatic and soil factors [5]. Weed management strategies play a vital role in successful crop production. Different weed management techniques are applied, such as cultural, physical, chemical, and biological techniques. Due to the scarcity of labor, the use of chemical herbicides or weedicides to decrease weed populations is becoming more popular throughout the world [6]. To control weeds, chemical methods are largely used in agriculture due to their easy application, cost effectiveness, and availability [7,8]. However, synthetic herbicidal weed control is hazardous to both the environment and people [9,10]. Furthermore, the long-term use of synthetic herbicides with limited variation in the mode of action has resulted in the emergence of herbicide resistance. Presently, more than 250 species of weeds have developed resistance around the world [11]. Based on these problems, many research studies have been conducted to investigate reducing the dependency on synthetic herbicides, and subsequently, biological weed control appears to be the most suitable alternative for herbicidal weed control [12,13]. This strategy involves using substances found in crop extracts and decomposed microbes [14,15].

*C. tenuis* Roxb. (local name bet in Bangladesh) is a shrub-type palm belonging to the Arecaceae family. It is an evergreen perennial plant with a slender stem and forms clusters [16]. This plant is found in India, Bangladesh, Thailand, Myanmar, Indonesia, and Cambodia [17], and is a predominant species in the northeastern part of Bangladesh. This plant species grows in foothills to plain slopes, riverbanks, and shady, damp, and wet areas [18]. They can also grow in weed-infested areas without any intercultural operations. The products of this forest plant are non-woody and have high economic value [19], very light, flexible, and durable features, and considered an important raw material for the cottage and handicraft industries. The young stems or the upper part of stems are used as both a vegetable and in traditional medicine [20] to treat fevers, dyspepsia, biliousness, piles, bacterial infections, wounds [21], diabetes [22], inflammation [23], stomach disorders, and intestinal infections [24]. Moreover, researchers have documented different biological activities of *C. tenuis*, including antioxidant, phytochemical, cytotoxic, and antibacterial activities, and may serve as a new potential source of medicines for humans [17,25,26]. Furthermore, Thakur et al. [27] found that methanol extracts of *C. tenuis* contain different secondary metabolites, including flavonoids, carbohydrates, saponins, glycosides, and steroids. Although different biological activities have been recorded for this species, no documents were found in the literature about the phytotoxic potential of this plant. Thus, the purpose of this research was to assess the phytotoxic effect of *C. tenuis* and to identify its phytotoxic compounds that may explain the dominant behavior of this plant species, and to provide information that could be helpful for developing new bioherbicides.

## 2. Results

### 2.1. Phytotoxicity of the C. tenuis Extract

**Figure 1:**
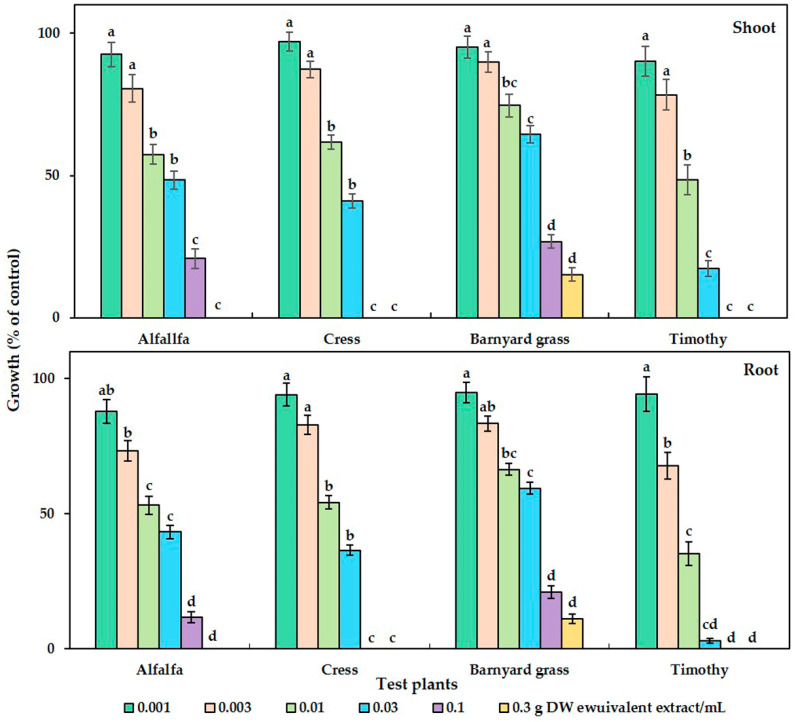
The growth of alfalfa (*Medicago sativa*), barnyard grass (*Echinochloa crus-galli*), cress *(Lepidium sativum*), and timothy (*Phleum pretense*) at different *C. tenuis* extract concentrations. The mean values, along with their standard errors, were obtained from two separate trials, each consisting of 3 replications. Vertical bars show the standard error. Different letters denote a 5% significant difference between the treatment and control.

The phytotoxicity of the *C. tenuis* aqueous methanol extract had a significant effect on all the tested plant species (Figure 1). Dose-dependent growth inhibitory effects in parallel with the increase in extract concentration were observed, regardless of the test plant species. Although no statistically significant growth inhibitory effect was observed with the initial dose of the *C. tenuis* extract (0.001 g DW/mL), its inhibitory activity was increased with higher concentrations. The seedling growth of timothy was inhibited by more than 50% at 0.01 g DW of *C. tenuis* extract/mL, and the shoot and root was inhibited by 57.33%, 61.67 %, and 74.61%, and 53.21%, 54.18 %, and 66.42% of the control in the cases of alfalfa, cress, and barnyard grass, respectively. Notably, the seedling growth rates of cress and timothy were fully suppressed by 0.1 g DW/mL of the *C. tenuis* extract, although alfalfa and barnyard grass were limited to less than 30% of the control treatment. All test plant seedlings except for barnyard grass were totally inhibited at 0.3 g DW/mL of the *C. tenuis* extract.

The concentration of *C. tenuis* leaf extract required to suppress 50% of the seedling growth (I_50_ values) of all the test plants was within the range of 5.78–47.57 mg DW equivalent *C. tenuis* extract/mL (Table 1). Timothy was the most sensitive species to the extract, whereas barnyard grass was the least sensitive, as indicated by their respective I_50_ values.

### 2.2. Isolation and Characterization of the Active Substances

**Figure 2:**
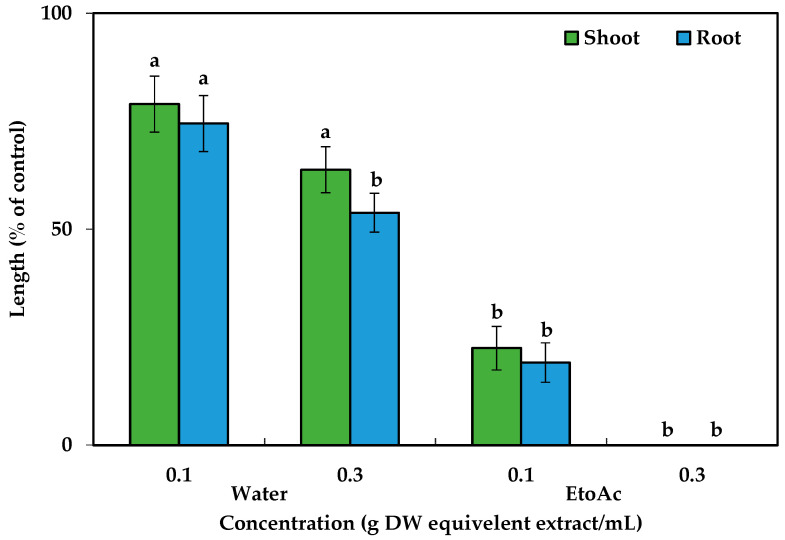
Cress responses to *C. tenuis* aqueous and ethyl acetate extracts. The mean values, along with their standard errors, were obtained from two separate trials. Vertical bars show the mean standard error. Different letters denote a 5% significant difference between the treatment and control.

**Figure 3:**
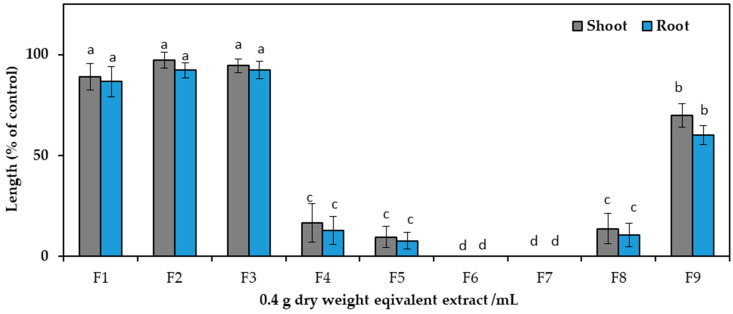
Effect of silica gel column fractions on the seedling growth of cress at the concentration 0.4 g dry weight equivalent extract/mL of *C. tenuis*. The values are mean ± SE obtained from two independent experiments. Vertical bars show the standard error. Different alphabet letters indicate significant differences between the treatment and control at a 5% probability level.

After dividing the aqueous methanol crude extracts, ethyl acetate and aqueous fractions were analyzed against cress seedlings at 0.1 g and 0.3 g DW equivalent *C. tenuis* extract/mL. Compared to the aqueous fraction, ethyl acetate exhibited greater phytotoxic activity (Figure 2) and was consequently chosen for the next isolation process and chromatographed on a silica gel column, yielding nine fractions through eluting with increasing amounts of ethyl acetate (10% per step, *v*/*v*) in n-hexane: F1, F2, F3, F4, F5, F6, and F7 contained 20%, 30%, 40%, 50%, 60%, 70%, and 80% ethyl acetate in n-hexane, respectively, followed by F8 (ethyl acetate), and F9 (methanol) (Figure 3). The column was among these fractions; the most phytotoxic activity was found from fractions six and seven obtained from 70% and 80% ethyl acetate/n-hexane.

These fractions were then purified with Sephadex LH-20, a reverse-phase C_18_ cartridge, and HPLC, yielding two pure compounds, substances 1 and 2. Finally, two purified substances were characterized through spectroscopic analysis. The NMR spectral data of substance 1 has been summarized in Table 2.

**Figure 4:**
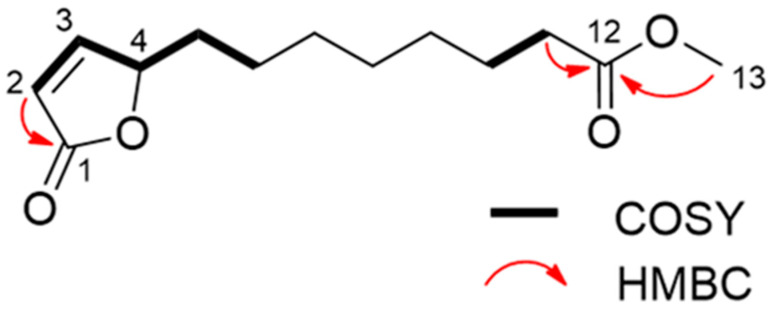
Gross structure of calamulactone ((*S*)-methyl 8-(5-oxo-2,5-dihydrofuran-2-yl) octanoate) via 1D and 2D NMR spectroscopy.

**Figure 5:**
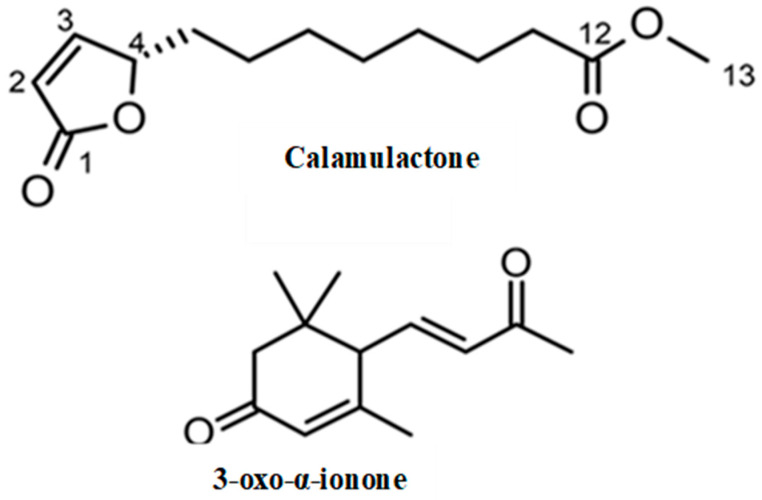
Molecular structures of calamulactone ((*S*)-methyl 8-(5-oxo-2,5-dihydrofuran-2-yl) octanoate) and 3-oxo-α-ionone from the *C. tenuis* leaf extracts.

Through spectroscopic analysis, the chemical formula of substance 1, a colorless oil, was determined to be C_13_H_20_O_4_ via HRESIMS. The NMR data for substance 1 has been summarized in Table 2. The ^1^H NMR spectrum of substance 1 indicated one methoxy group (singlet at *δ*_H_ 3.67). The ^13^C NMR spectrum suggested two carbonyl carbons (*δ*_C_ 174.4 and 173.3) and two olefinic carbons (*δ*_C_ 156.3 and 121.8). The remaining carbon signals were assigned to one methoxy group, seven methylene groups, and one methine based on the results of an HSQC experiment. The gross structure was determined based on 1D and 2D NMR experiments (Figure 4). A detailed analysis of the COSY and HSQC spectra of substance 1 allowed for the identification of two partial structures, C-2 to C-6 and C-10 to C-11. The HMBC correlations between H-2/C-1 and H-11/C-12 suggested the connectivity of C-1-C-2 and C-11-C-12. The linkage of C-1 to C-4 via an oxygen atom (O_2_) was confirmed through the chemical shifts consistent with an α, β-unsaturated γ-lactone ring [28]. In addition, the HMBC correlation between H-13/C-12 indicated that the methoxycarbonyl part was joined to C-12. The connectivity of C-6-C-10 was confirmed through its molecular formula and degree of unsaturation. Hence, the gross structure of substance 1 was determined to be as shown in Figure 5. The absolute configuration of C-4 was determined on the basis of the ECD spectrum according to a previous report [29]. As the ECD spectra of substance 1 showed a positive π-π* transition at 204 nm, the configuration of C-4 was determined to be 4S. Thus, we assigned substance 1 as a novel compound, (*S*)-methyl 8-(5-oxo-2,5-dihydrofuran-2-yl) octanoate (calamulactone). On the other hand, the chemical structure of substance 2 was determined as 3-oxo-α-ionone through its ^1^H NMR spectrum as measured in CDCl_3_ in comparison with reported data [30]. The molecular structures of both substances are shown in Figure 5.

### 2.3. Phytotoxicity of the Identified Substances

**Figure 6:**
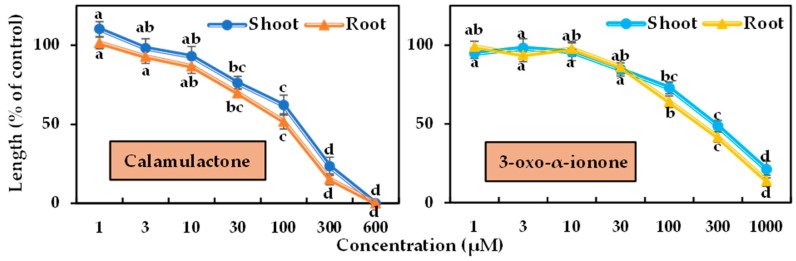
Phytotoxic effects of calamulactone and 3-oxo-α-ionone on cress. Values are the means ± SE from three replications. Different letters denote statistically significant variations between the treatment and control groups.

**Figure 7:**
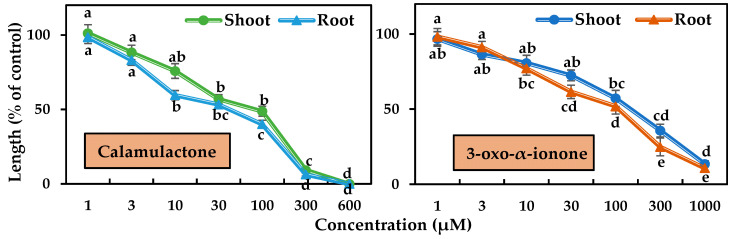
Phytotoxic effects of calamulactone and 3-oxo-α-ionone on timothy. Values are the means ± SE from three replications. Different letters denote statistically significant variations between the treatment and control groups.

The phytotoxicity of the two isolated compounds was tested in a bioassay using cress and timothy at varying doses. Differences in phytotoxicity were observed between the two tested plants’ seedling growth, and the magnitude of phytotoxicity increased with increasing doses (Figure 6 and Figure 7). Calamulactone and 3-oxo-α-ionone started to significantly limit the seedling growth of the cress at the concentration of 30 μM. At 100 μM, calamulactone inhibited cress shoot growth by 62.14% and root growth by 51.80% from the control, while 3-oxo-α-ionone reduced the growth of cress shoots and roots to 72.89% and 64.10%, respectively. At higher concentrations, calamulactone became more toxic and completely inhibited cress growth at 600 μM. However, at 1000 μM, 3-oxo-α-ionone inhibited cress growth to just 21.02 %and 13.96% of the control in the shoot and root, respectively.

In the case of timothy, both compounds started to suppress the seedling growth at 10 μM. Calamulactone, at 100 μM, inhibited shoot growth by 48.79% and root growth by 40.10%, respectively, while 3-oxo-α-ionone had a comparatively less phytotoxic effect and suppressed shoot growth by 57.20% and root growth by 51.90%, respectively. At the higher concentration of 600 μM, calamulactone completely stopped timothy seedling growth, while at 1000 μM, 3-oxo-α-ionone restricted the shoot by 13.58% and the root by 10.74% of timothy seedlings of control, respectively.

Calamulactone and 3-oxo-α-ionone suppressed cress seedling growth by 50% (I_50_) at concentrations of 105.6–141.1 μM and 199.2–281.6 μM, respectively, where the inhibitory effect of calamulactone was clearly more pronounced than 3-oxo-α-ionone (Table 3). Similarly, in the case of timothy, 3-oxo-α-ionone inhibited growth by 50% at concentrations within the range of 107.8–144.7 μM, and calamulactone induced the same inhibitory effect at only 40.5–83.4 μM. Moreover, the root growth of both tested plants was significantly more affected by both substances compared with the corresponding shoot growth (Table 3).

## 3. Discussion

The current investigation found that the *C. tenuis* leaf extracts (aqueous methanol) strongly inhibited alfalfa, barnyard grass, cress, and timothy growth in a dose-dependent manner proportional to treatment concentration. Our findings corroborate the findings of several studies reporting the concentration-dependent allelopathic effects of different plant species extracts against several monocot and dicot test plants [31,32,33]. Moreover, different test plants showed different levels of sensitivity to the *C. tenuis* extracts. Some researchers have postulated that metabolic systems in different plant species become disordered when the concentration of a leaf extract exceeds a threshold, which is linked to the inherent tolerance mechanisms of the plants [34]. Other researches have also documented the species-specific phytotoxic action of plant extracts [35,36].

The extracts of *C. tenuis* may have a growth-inhibiting effect, as they may contain growth-limiting substances that interfere with the target species’ numerous physiological processes [37]. The isolation and identification of active compounds are one of the most vital areas of research in developing eco-friendly bioherbicides [38]. Thus, the current research aimed to isolate bioactive substances from the *C. tenuis* leaf extracts through chromatographic fractionations, with the highest active fraction containing a higher number of phytotoxic substances, imparting a higher level of phytotoxicity. Two bioactive substances 1 and 2, were isolated from the *C. tenuis* extracts.

The bioassays of calamulactone and 3-oxo-α-ionone against cress and timothy revealed strong dose-dependent phytotoxicity (Figure 3 and Figure 4). Many recent studies have shown such a concentration-dependent growth-limiting effect of plants containing phytotoxic substances [39,40,41,42,43,44]. Comparing I_50_ values, calamulactone suppressed cress shoot growth 1.99 times more than 3-oxo-α-ionone, and root growth 1.89 times more. Calamulactone, like 3-oxo-α-ionone, inhibited timothy growth, but at a 1.71- and 2.65-fold higher rate in the shoot and root, respectively. Both compounds had a more pronounced effect on the seedling growth of timothy than cress. Species specificity is demonstrated by the fact that various plant species have varied reactions to the same phytotoxic chemicals. This is due to the unique physiological and biochemical characteristics of each plant species [45]. Phytotoxic chemicals affect the early seedling growth of test plants, mostly through seed size and coat [46]. Large seeds with a prominent seed coat are less susceptible to phytotoxic substances than smaller seeds. The seed size of cress is almost five times higher than that of timothy, and consequently, it is less sensitive to both substances. These results corroborate our earlier observations that timothy is more susceptible to the toxic effects of a variety of plant metabolites [40,47].

Root growth was significantly more susceptible to the investigated substances than shoot growth in both plant species, which has been proposed as the best indicator of phytotoxicity of any growth inhibitory substance [34,48]. This finding can be explained by the roots being the first organ to emerge from a plant, and consequently, root tissues are the first to come into contact with the allelochemicals in the moist filter paper, resulting in the roots absorbing more allelochemicals than the shoots [49,50,51], which in turn leads to a limited division of cells [52] and restricts root growth.

In terms of phytotoxicity, calamulactone showed higher inhibitory activity than 3-oxo-α-ionone. Calamulactone is a derivative of an α, β-unsaturated γ-lactone. Although this report is the first on calamulactone as a novel compound, the derivatives of α, β-unsaturated γ-lactone are widely found in nature from plants, fungi, and animal sources [53]. It has been reported that compounds having a γ-lactone moiety may play a vital role in chemical defense systems [53,54]. Some of these substances exhibit different biological activities, including insecticidal, antibacterial, phytotoxic, and antifungal activities [55]. The γ-lactone rings of these substances have different patterns of saturation and substitution, and most are attached with a large carbon skeleton. In general, α, β-unsaturated γ-lactones show better phytotoxic activities than saturated ones [54]. On the other hand, 3-oxo-α-ionone is a carotenoid-derived anorisoprenoid [56]. This substance has several medicinal features, and its phytotoxicity is also well known. The allelopathic effects of the 3-oxo-α-ionone compound have been shown by different researchers and identified from many plant species, such as *Albizia richardiana* [57], *rattail fescue* [58], *Withania aristate* [59], and *Vallisneria spiralis* [60]. Although the phytotoxicity of this substance has been well documented, this report is the first on the isolation of 3-oxo-α-ionone from *C. tenuis*. Thus, structural variations between these two compounds might be the reason for their differential phytotoxic behavior. Moreover, calamulactone and 3-oxo-α-ionone likely account for at least a significant part of *C. tenuis* phytotoxicity. The strong phytotoxicity of *C. tenuis* may explain its dominance over surrounding plant species, which enables it to grow vigorously, even in places with considerable weed pressure.

Bioherbicides are regarded as a vital tool for the management of weeds, as they are developed from the extracts of different plants. Bioherbicides showed a promising influence on different weeds. When bioherbicides are applied in the field, they affect the germination, growth, and development of different weed species. Usually, they do not have a long residual effect, are not harmful for the soil and water, and do not affect the non-target organisms. Thus, bioherbicides could be used as an environment friendly weed management method. In Bangladesh, *C. tenuis* plant species are available in the northeastern region; the results of this study provide suitable information about its phytotoxicity, which will be helpful for the farmers to apply this species for weed management.

## 4. Conclusions

The current study revealed the strong phytotoxicity of aqueous extracts of *C. tenuis* against the seedling growth of monocotyledonous and dicotyledonous standard test plants. Bioassay-directed purification yielded two substances, a novel compound, calamulactone, and a known substance, 3-oxo-α-ionone. Both substances showed significant phytotoxicity against cress and timothy. In particular, with its low I_50_ values, calamulactone showed notable phytotoxicity against the test plants. The phytotoxicity of the *C. tenuis* extracts and its phytotoxic substances may play a crucial role in the defense and survival mechanisms of this species. In addition, this study provides indications for eco-friendly weed management.

## 5. Materials and Methods

### 5.1. Plant Samples

Fresh, healthy *C. tenuis* Roxb. leaves were obtained around Sylhet Agricultural University (24.8917° N 91.8833° E) from May to June in the year 2017. The leaves were washed with purified water to get rid of dirt, dust, and other surface impurities. They were then left to dry in the shade for 15 days, and an electric blender machine was used to grind them into a coarse powder. The powder was put in a thick plastic bag and kept at 2 °C until it was used. Four test plant species were selected for phytotoxicity assays: two monocots (*Echinochloa crus-galli* (L.) P. Beauv. (barnyard grass) and *Phleum pratense* L. (timothy)) and two dicots (*Medicago sativa* L. (alfalfa) and *Lepidium sativum* L. (cress)).

### 5.2. Extraction and Bioassay

An initial extraction was carried out to check the phytotoxicity of the plant materials and to design a precise isolation process. Accordingly, 100 g of *C. tenuis* leaves were extracted via soaking in 500 mL of methanol (70% methanol) in a dark condition for two days and filtered through filter paper (one layer, No. 2, 125 mm; Toyo Ltd., Tokyo, Japan). After a day of dissolving the solids in an equivalent volume of methanol, they were filtered once again. A Rotavapor was used to dry the combined extracts at 40 °C. Filter paper (No. 2, 28 mm; Toyo) was placed in Petri plates with a final concentration of 0.001, 0.003, 0.01, 0.03, 0.1, and 0.3 g of DW (dry weight) equivalent extract/mL, which were prepared through dissolving the extracts in 100 mL of methanol. After the methanol was removed via evaporation, 0.6 mL of a 0.05% aqueous solution of Tween 20 (polyoxyethylene sorbitan monolaurate; Nacalai Tesque, Inc., Kyoto, Japan) was added to each Petri dish to moisten the surface. Each Petri plate received ten homogeneous and ten pre-emergent seeds of dicots and monocots, respectively. The control was aqueous Tween 20-treated Petri dishes without *C. tenuis* extracts. After two days in a germinator at 25 °C in the dark, all Petri dishes were measured for seedling length.

### 5.3. Purification of the Active Substances

A separate extraction was carried out to identify any bioactive compounds. *C. tenuis* powder (2 kg) from the same sample source was subjected to the above-described extraction procedure, and the solvent of the produced extracts was removed using the Rotavapor (40 °C) to obtain an aqueous methanol crude extract. Following neutralization at pH 7.0 with 1 M phosphate buffer, the crude extract was partitioned four times with the same volume of ethyl acetate. Cress was used in bioassays for both the aqueous and ethyl acetate extracts. The bioactive substances were isolated and identified from the ethyl acetate fraction, since it demonstrated the highest activity. After eliminating the undesirable water with anhydrous Na_2_SO_4_, the ethyl acetate fraction was dried, separated on a silica gel column (60 g of silica gel 60, spherical, 70–230 mesh; Nacalai Tesque), and eluted with an ethyl acetate and n-hexane mixture (from 20% to 100%, increased 10% per step) and methanol. Based on the result of the bioassay, the strongest activity was obtained from the 70% and 80% ethyl acetate in *n*-hexane fractions. Purification of both fractions was accomplished by passing them over a Sephadex LH-20 column (GE Healthcare Bio-Sciences AB, SE-751 84, Uppsala, Sweden) and fractionating them at increasing methanol concentrations from 20% to 100%. The most impressive result was achieved with 40% aqueous methanol, which was then fractionated with a C18 reverse-phase cartridge. Eluting the cartridge with 20–80% methanol in water and methanol resulted in five distinct fractions. The highest level of activity was recorded using a solution consisting of 40% methanol in water. This solution was purified using reverse-phase high-performance liquid chromatography (HPLC) with a column of dimensions 500 × 10 mm (inner diameter) and ODS AQ-325 packing material, manufactured by YMC Ltd. in Kyoto, Japan. The purification process was carried out at a flow rate of 1.5 mL/min. The composition of the mobile phase consisted of a 40% aqueous methanol solution. The chromatogram was obtained through recording the absorbance at a wavelength of 220 nm at a temperature of 40 °C. The two most active substances, 1 and 2, had retention periods of 80–90 min and 130–140 min, respectively. To enhance purification, a precise reverse-phase HPLC system (4.6 × 250 mm I.D., S-5 µm, Inertsil^®^ ODS-3; GL Science Inc., Tokyo, Japan) was used. A 30% aqueous methanol mobile phase was used to purify at 0.8 mL/min and isolated substances 1 and 2 at 35–40 and 60–70 min, respectively. Finally, substances 1 and 2 were identified using different spectroscopic analyses: HRESIMS, ^1^H-NMR, and ^13^C-NMR.

### 5.4. Characterization of the Compounds

#### Spectral Data

Electronic circular dichroism (ECD) spectra were recorded on a JASCO J-820 spectropolarimeter (JASCO, Tokyo, Japan). The Bruker AVANCE III was used to collect ^1^H (500 MHz) and ^13^C (125 MHz) NMR spectroscopic data. There were observed chemical shifts in comparison to the residual solvent signal (CDCl_3_: *δ*_H_ 7.26, *δ*_C_ 77.16). The Waters Micromass Q-TOF spectrometer (Waters Corporation, Milford, MA, USA) was used to record the HRESIMS data.

Calamulactone: colorless oil; ECD (MeOH) *λ*_ext_ 204 nm, ∆*ε* +3.65; ^1^H NMR (500 MHz, CDCl_3_) *δ_H_* 7.44 (dd, *J* = 1.5, 5.7, H-3, 1H), 6.11 (dd, *J* = 2.0, 5.7, H-2, 1H), 5.03 (m, H-4, 1H), 3.67 (s, 3H, H-13), 2.30 (t, *J* = 7.4, 2H, H-11), 1.76 (m, 1H, H-5a), 1.66 (m, 1H, H-5b), 1.62 (m, 2H, H-10), 1.44 (m, 2H, H-6), and 1.32 (m, 6H, H-7, H-8, and H-9); ^13^C NMR (125 MHz, CDCl_3_) *δ*_C_ 174.4, 173.3, 156.3, 121.8, 83.5, 51.6, 34.2, 33.3, 29.22, 29.12, 29.08, 25.04, and 24.98; HRESIMS *m/z* 241.1441 [M + H]^+^ (calcd for C_13_H_21_O_4_, 241.1440).

3-oxo-α-ionone: ^1^H NMR (CDCl_3_): 1.05 (s, 1-CH3, 3H), 1.11 (s, 1-CH3, 3H), 1.94 (d, 5-CH3,3H), 2.12 (d, 1H, *J* = 16 Hz, 2-H), 2.40 (d, 1H, *J* = 16 Hz, 2-H), 2.32 (s, 9-CH_3_, 3H), 2.78 (d, 1H, *J* = 9 Hz, 6-H), 6.04 (s, 1H, 4-H), and 6.18 (d, 1H, *J* = 15.5 Hz, 8-H), 6.72 (dd, 1H, *J* = 9 Hz, *J* = 15.5 Hz, 7-H); HRESIMS *m/z* 207.1307 [M + H]^+^ (calcd for C_13_H_18_O_2_, 206.1307) [41].

### 5.5. Bioassay of Calamulactone and 3-Oxo-α-Ionone

Using the same bioassay approach described before, the bioactivity of calamulactone and 3-oxo-α-ionone was evaluated against cress and timothy at doses of 1 µM, 3 µM, 10 µM, 30 µM, 100 µM, 300 µM, 600 µM, and 1000 µM dissolved in methanol.

### 5.6. Statistical Analysis

The assay was performed in triplicate and then replicated twice using a CRBD. The data were presented as means and standard errors. One-way analysis of variance (ANOVA) with Tukey’s post-hoc test was used to determine statistical significance between the treatment and control groups in SPSS (20.0). Statistical significance was defined as a *p*-value of 0.05. Data was reported as a percentage of the control group. In the assay experiments, GraphPad Prism 6.0 (GraphPad Software, Inc., La Jolla, CA, USA) was used to calculate species I_50_ values.

## Figures and Tables

**Table 1:** *C. tenuis* extract doses that inhibit test plant seedling growth by 50% (I_50_).

Test Plant Species		*C. tenuis* Aqueous Methanol Extract (mg DW/mL)	
		Shoot	Root
Dicotyledonous	Alfalfa	24.60	14.24
	Cress	18.65	12.97
Monocotyledonous	Barnyard grass	47.57	40.33
	Timothy	9.42	5.78

**Table 2:** NMR spectral data for substance 1 in CDCl_3_.

Position	*δ*_H_ Mult (*J* in Hz) ^a^	*δ*_C_ ^b^
1		173.3
2	6.11, dd (5.7, 2.0)	121.8
3	7.44, dd (5.7, 1.5)	156.3
4	5.03, m	83.5
5a	1.76, m	33.3
5b	1.66, m	
6	1.44, m	25.04
7	1.32, m ^c^	29.22
8	1.32, m ^c^	29.12
9	1.32, m ^c^	29.08
10	1.62, m	24.98
11	2.30, t (7.4)	34.2
12		174.4
13	3.67, s	51.6

^a^ Recorded at 500 MHz. ^b^ Recorded at 125 MHz. ^c^ Overlapped signals.

**Table 3:** I_50_ values of calamulactone and 3-oxo-α-ionone for cress and timothy.

Test Plant		Calamulactone	3-Oxo-α-Ionone
		(µM)	
Cress	Shoot ***	141.1	281.6
	Root ***	105.6	199.2
Timothy	Shoot ***	83.4	144.7
	Root ***	40.5	107.8

A significant difference in shoot and root growth across test plants is indicated by *** *p* < 0.001 (pair-wise *t*-test).

## Data Availability

All data from this study are included within this manuscript.
